# Brain Structure Alterations in Respect to Tobacco Consumption and Nicotine Dependence: A Comparative Voxel-Based Morphometry Study

**DOI:** 10.3389/fnana.2018.00043

**Published:** 2018-05-24

**Authors:** Peng Peng, Min Li, Han Liu, Ya-Ru Tian, Shui-Lian Chu, Nicholas Van Halm-Lutterodt, Bin Jing, Tao Jiang

**Affiliations:** ^1^Department of Radiology, Beijing Chaoyang Hospital, Capital Medical University, Beijing, China; ^2^School of Biomedical Engineering, Capital Medical University, Beijing, China; ^3^Clinical Research Center, Beijing Chaoyang Hospital, Capital Medical University, Beijing, China; ^4^Department of Neurosurgery, Beijing Tiantan Hospital, Capital Medical University, Beijing, China; ^5^Department of Orthopaedics and Neurosurgery, Keck Medical Center of USC, University of Southern California, Los Angeles, CA, United States

**Keywords:** smoking, voxel-based morphometry, nicotine dependence, pack-years, FTND score

## Abstract

The main purpose of this study is to examine the lifetime tobacco consumption and the degree of nicotine dependence related gray matter (GM) and white matter (WM) volume alterations in young adult-male smokers. Fifty-three long-term male smokers and 53 well-matched male healthy non-smokers participated in the study, and the smokers were respectively categorized into light and heavy tobacco consumption subgroups by pack-years and into moderate and severe nicotine dependence subgroups using the Fagerström Test for Nicotine Dependence (FTND). Voxel-based morphometry analysis was then performed, and ANCOVA analysis combined with subsequent *post hoc* test were used to explore the between-group brain volume abnormalities related to the smoking amount and nicotine dependence. Light and heavy smokers displayed smaller GM and WM volumes than non-smokers, while heavy smokers were found with more significant brain atrophy than light smokers in GM areas of precuneus, inferior and middle frontal gyrus, superior temporal gyrus, cerebellum anterior lobe and insula, and in WM areas of cerebellum anterior lobe. However, the contrary trend was observed regarding alterations associated with severity of nicotine dependence. Severe nicotine dependence smokers rather demonstrated less atrophy levels compared to moderate nicotine dependence smokers, especially in GM areas of precuneus, superior and middle temporal gyrus, middle occipital gyrus, posterior cingulate and insula, and in WM areas of precuneus, posterior cingulate, cerebellum anterior lobe and midbrain. The results reveal that the nicotine dependence displays a dissimilar effect on the brain volume in comparison to the cigarette consumption. Our study could provide new evidences to understand the adverse effects of smoking on the brain structure, which is helpful for further treatment of smokers.

## Introduction

Smoking, which is one of the most critical and preventable causes of morbidity and mortality, has always been a thriving focus in research studies. With the development of magnetic resonance imaging (MRI), it has rapidly evolved into an unrivaled tool for the assessment and evaluation of brain activity and structural changes *in vivo*. A rising number of functional MRI studies have been conducted to examine the effects of acute nicotine intake between smokers and non-smokers, and a common finding is that, nicotine globally mitigates brain activities (Brody, 2006; Jasinska et al., 2014). In addition, alterations in brain activities during the resting state functional MRI in chronic smokers have also been comprehensively reported (Ding and Lee, 2013; Yu et al., 2013; Feng et al., 2016). Generally speaking, functional MRI is possibly influenced by some latent factors, which leads to a not very high reproducibility and reliability, limiting its usage in clinical applications.

Voxel-based morphometry (VBM) (Ashburner and Friston, 2000) is a widely used method to study brain structural damages or changes. More recently, the adverse effects of smoking on brain structural changes have received substantial focus (Durazzo et al., 2013; Fritz et al., 2014), with some interesting studies reporting outcomes in the relationships that exist between smoking and thickness or density of cerebral cortex (Yu et al., 2013; Karama et al., 2015; Li et al., 2015; Power et al., 2015). A previously reported study focused on the volume changes of specific brain regions of interest, as drawn by hand, but not of whole-brain GM or WM (Prom-Wormley et al., 2015), while another study reported the effects of multiple addictive factors on structural changes in the brain (Pennington et al., 2015). Additionally, some studies researched whole-brain volume changes (Durazzo et al., 2012; Hoogendam et al., 2012), irrespective of changes belonging to either gray matter (GM) or white matter (WM). However, GM and WM display different physiological functions, with tobacco potentially exerting different pathophysiological effects on them. Therefore, changes caused by cigarette should be separately studied in whole-brain GM or WM. To date, only a few studies have explored the effects of tobacco on GM and WM separately (Ikram et al., 2008; Duriez et al., 2014; Wang et al., 2015).

It is also critical to emphasize that different kinds of smoking populations were selected by researchers. Generally, middle-aged and aged adults were adopted in most studies (Ikram et al., 2008; Duriez et al., 2014; Karama et al., 2015; Pennington et al., 2015; Prom-Wormley et al., 2015), but some other studies also focused on adolescent/young adult smokers or smokers with different gender ratios (Prom-Wormley et al., 2015). These studies obtained inconsistent or even opposite results, indicating age-specific and gender-specific effects of nicotine on the brain of smokers. Therefore, these influential factors should be controlled as far as possible especially in studies of comparing different factors’ effects on smokers.

Smoking account and nicotine dependence are two independent factors on smokers, It is well accepted that more smoking account could cumulatively lead to more serious brain alternations, and our previous study had reported the structural changes in the brain related to the lifetime tobacco consumption (in pack-years) (Peng et al., 2015). However, whether nicotine dependence also displays such a trend as smoking account is still unknown for smokers, and we speculate nicotine dependence may have distinct effects on the brain compared to the smoking account. To elucidate the question in this study, the whole-brain GM/WM volume alterations induced by nicotine dependence were compared in the same population of smokers in our previous study. To the best of our knowledge, the effects of smoking amounts and nicotine dependence on brain structure have not been comprehensively compared using a relatively large sample size. Therefore, the objectives of the current study are: (i) to reveal the whole-brain GM/WM volume alterations in the brain related to the nicotine dependence; (ii) to determine whether the lifetime tobacco consumption and nicotine dependence impose similar change trend on the brain structure. Specifically, only young adult male smokers were enrolled in the study since they substantially contribute to a major fraction of the smoking population in China as well as maximizing the control of confounding factors that may influence brain structural outcomes associated with age and gender differences.

## Materials and Methods

### Subjects

The institutional review board of Beijing Chaoyang Hospital approved this study. We recruited 53 young male smokers and 53 age-matched healthy non-smokers as controls. All participants provided their written informed consents to participate in this study. All subjects were Han Chinese male persons aged from 23 to 40 years with an undergraduate education or higher. A routine head MRI scan was initially performed on each subject to ensure that the participant didn’t display obvious structural abnormalities in the brain.

We performed semi-structured telephone interviews to collect and assess the participants’ smoking, medical, psychiatric, medication, and substance usage information. The inclusion criteria for smokers were: (1) smoked 5 or more cigarettes per day during the previous year; (2) smoked more than 5 years and had not abstained from smoking for longer than 3 months in the past year; and (3) satisfied the International Classification of Diseases (ICD)-10 criteria for nicotine dependence (World Health Organization [WHO], 1992). We administered the same questionnaire designed for the use in the 2010 Global Adult Tobacco Survey (GATS) by the World Health Organization (WHO), which included the age at which one began smoking, the number of cigarettes smoked per day, the years of smoking, duration of smoking cessation, and so forth. The Fagerström Test for Nicotine Dependence (FTND) was also administered to evaluate the severity of nicotine dependence for each smoker. A FTND score of ≤6 represents moderate dependence, and a FTND score of >6 indicates severe dependence. Based on the FTND score, the smokers were separated into moderate and severe dependence groups. Pack-years represents another commonly used parameter in clinic to quantitatively characterize smokers’ lifetime tobacco consumption. The number of pack-years is calculated as follows: (packs smoked per day) × (years as a smoker) or (number of cigarettes smoked per day) × (years as a smoker)/20 (where 1 pack of cigarette contains 20 cigarettes). This measure is an effective indicator of smoking hazard exposure to people, and it’s been proven to be associated with the incidence of various diseases, such as cancers, cognitive abnormalities, cardiovascular diseases and so on. However, there is currently no definite standard to differentiate between light and heavy tobacco consumption smokers, and previous studies have grouped smokers based on the subjects’ median, tertile, or quartile pack-years (Cena et al., 2013; Yuan et al., 2017). Accordingly, we separated smokers into categories of light and heavy tobacco consumption smokers by the median pack-years (median = 21), and some researchers have found that a tobacco consumption more than 20 pack-years could significantly increase the risk of certain diseases (Janjigian et al., 2010; McEvoy et al., 2015). Non-smokers were defined as individuals who had never smoked any cigarette prior to this study.

The exclusion criteria for all groups were: (1) age of less than 18 years; (2) any current or past diagnosis of multiple sclerosis, schizophrenia, Parkinson’s disease, Alzheimer’s disease or any other diseases of the central nervous system that may influence outcomes in cognitive performances or brain structure; (3) any current or past diagnosis of alcohol or drug abuse/dependence; (4) any history of head injury, such as skull fracture, brain tumor, or brain surgery; (5) any current or previous psychiatric disorders or usages of any psychotropic medications; (6) a family history of psychotic disorders; and (7) any other contraindications for MRI.

### MRI Data Acquisition

All MRI data were obtained using a 3.0T scanner (MAGNETOM^®^ Trio Tim System; Siemens Medical Solutions, Erlangen, Germany) with a standard 8-channel head coil receiver at the magnetic resonance center of Beijing Chaoyang Hospital. All participants were asked to relax and remain as still as possible during the scanning, and foam pads were used to restrict the head movements. Three-dimensional whole-brain high-resolution T1-weighted images (T1WI-3D MPRAGE) were acquired using the following parameters: repetition time = 1900 ms; echo time = 2.52 ms; total slices = 176; slice thickness = 1 mm; matrix = 256 × 256; flip angle = 9°; in-plane voxel size = 1 mm × 1 mm × 1 mm; total acquisition time = 340 s.

### Voxel-Based Morphometry (VBM)

The data were processed using the Statistical Parametric Mapping software (SPM8) (Wellcome Department of Imaging Neuroscience Group, University College London, London, United Kingdom)^1^, by which we implemented VBM analysis using the DARTEL Tool with the default parameters^2^ pages: 197–202. The images were processed with the following steps: bias correction, tissue segmentation, and data registration using linear (12-parameter affine) and non-linear transformations (warping). Analyses were subsequently performed on the GM and WM segments. Finally, the normalized modulated volumes (voxel size: 1.5 mm) were smoothed using a Gaussian kernel with a full width at half maximum of 6 mm.

### Statistical Analyses

In the statistical analysis, we first separated smokers into light and heavy smokers using median pack-years (median = 21). The light smokers group consisted of 26 subjects (pack-years ranging from 2.5 to 19.5), and the heavy smokers group included 27 smokers (pack-years ranging from 21 to 48). Then, we regrouped smokers as moderate or severe dependence using the FTND score. There were a total of 23 smokers with moderate dependence (FTND score ranging from 1 to 5), while there were 30 smokers with severe dependence (FTND score ranging from 7 to 10). After that, we separately did the ANCOVA analysis among smokers with different smoking accounts (i.e., heavy smokers, light smokers, non-smokers) by setting age, intracranial volume, FTND as covariates, and among different nicotine dependence smokers (i.e., severe dependence smokers, moderate dependence smokers, non-smokers) by setting age, intracranial volume and pack-years as covariates. After the ANCOVA analysis, the between-group differences were subsequently obtained using the *post hoc* test.

The statistical significance has been specifically concerned since a previous famous paper (Eklund et al., 2016). The bug-corrected Alphasim correction is still an effective multiple comparison correction method, and could significantly reduce the false positives than non-corrected statistical method. The key idea behind the Alphasim correction is the permutation test, which is widely used in many fields and seems more applicable than the FWE/FDR correction (maybe too strict) for sMRI data. Therefore, Alphasim correction but not FWE/FDR corrections was adopted in the study, and an individual threshold of *p* < 0.005 and cluster size >42 was set corresponding to a *p* < 0.05 AlphaSim correction with the estimated smoothness. The statistical analysis was conducted with the DPABI software (V2.3)^3^.

### Correlation Analysis

To test whether the altered brain structures were significantly associated with tobacco consumption and nicotine dependence, every cluster from the between-group differences (i.e., heavy vs. light smokers, moderate vs. severe nicotine dependence) were selected as the candidate region, and partial correlation analysis was used to find the relationship between the pack-years/FTND score and the abnormal region volume with age, intracranial volume, FTND/pack-years as covariates.

## Results

The smoker and non-smoker groups were similar in age (mean: 30.72 ± 4.19 years vs. 30.83 ± 5.18 years, *p* = 0.911), and there were no significant differences in age and education level among smokers with different levels of tobacco consumption and non-smokers and among smokers with different degrees of dependence and non-smokers (*p* > 0.05, **Table 1**), while daily smoking amount, pack-years and FTND score were significantly different (*p* < 0.05). Notably, there were some *overlapping subjects* grouped by smoking amount and nicotine dependence, and a total of 12 light smokers had severe nicotine dependence (mean age: 27.08 ± 3.48 years) and 9 heavy smokers were found with moderate dependence (mean age: 34.78 ± 3.83 years).

**Table 1:** Demographic characteristics of smokers and non-smokers in the study.

	Age (years)	Levels of education (years)	Cigarettes per day	Age at start of smoking (years)	Pack-years^a^	FTND^b^ score
Smokers (*n* = 53)	30.72 ± 4.19	19.21 ± 1.39	22.51 ± 8.34	19.04 ± 3.94	20.51 ± 8.15	6.02 ± 2.63
Non-smokers (*n* = 53)	30.83 ± 5.18	19.32 ± 1.29				
*p*-value	0.911	0.679				
Light smokers (*n* = 26)	29.42 ± 4.43	19.12 ± 1.48	16.15 ± 5.16	18.50 ± 3.39	8.77 ± 3.57	5.15 ± 2.82
Heavy smokers (*n* = 27)	32.26 ± 3.73	19.30 ± 1.32	38.70 ± 8.36	19.56 ± 4.41	31.06 ± 7.40	6.85 ± 2.18
Non-smokers (*n* = 53)	30.83 ± 5.18	19.33 ± 1.29				
*p*-value	0.112^c^	0.816^c^	<0.001	0.334	<0.001	0.017
Mild/moderate addiction (*n* = 23)	32.74 ± 4.34	19.17 ± 1.80	18.07 ± 6.99	19.26 ± 3.36	15.93 ± 10.57	3.43 ± 1.65
Severe addiction (*n* = 30)	30.70 ± 4.86	19.26 ± 1.63	34.97 ± 7.31	18.87 ± 4.38	23.34 ± 13.33	8.00 ± 1.02
Non-smokers (*n* = 53)	30.83 ± 5.18	19.33 ± 1.29				
*p*-value	0.581^c^	0.925^c^	<0.001	0.722	0.033	< 0.001

*Values are expressed as means ± standard deviations. ^a^Pack-years: smoking years × daily consumption/20. ^b^FTND score: score of Fagerström Test for Nicotine Dependence. ^c^Statistical difference comparison among three groups (one-way ANOVA).*

**FIGURE 1:**
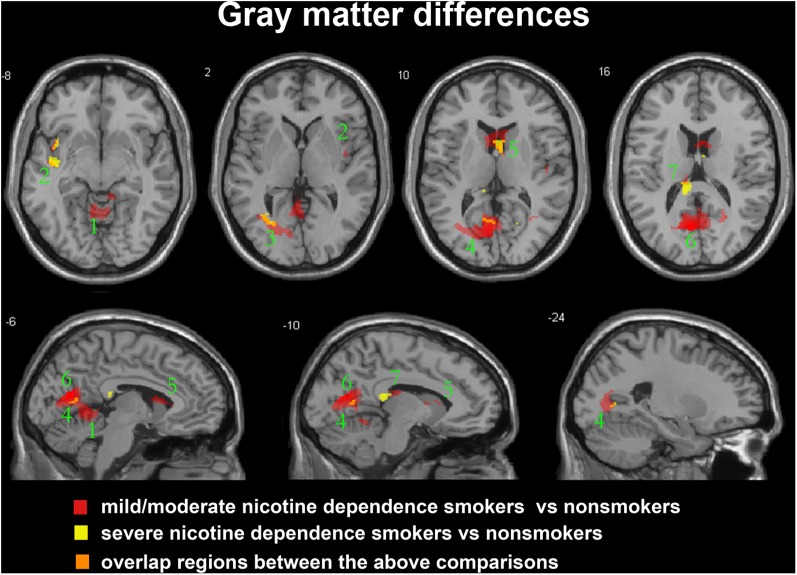
Regions with smaller gray matter volumes in 23 moderate nicotine dependence smokers and 30 severe nicotine dependence smokers than in non-smokers. This image shows the overlap (orange) regions of the smaller gray matter in 23 moderate nicotine dependence smokers (red) and 30 severe nicotine dependence smokers (yellow) than in 53 nonsmokers. Overall thresholds of significance were set at *P* < 0.05 (AlphaSim corrected). The **(top row)** shows four axial slices, whereas the **(bottom row)** shows three sagittal slices. 1, cerebellum anterior lobe; 2, insula; 3, middle occipital gyrus; 4, posterior cingulate; 5, caudate body; 6, precuneus; 7, thalamus.

**FIGURE 2:**
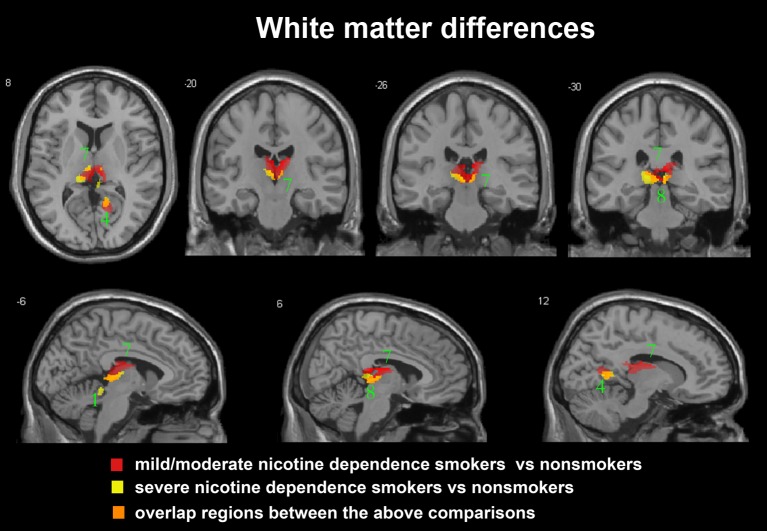
Regions with smaller white matter volumes in 23 moderate nicotine dependence smokers and 30 severe nicotine dependence smokers than in non-smokers. This image shows the overlap (orange) regions of the smaller white matter in 23 moderate nicotine dependence smokers (red) and 30 severe nicotine dependence smokers (yellow) than in 53 non-smokers. Overall thresholds of significance were set at *P* < 0.05 (AlphaSim corrected). The **(top row)** shows one axial slice and three coronal slices, whereas the **(bottom row)** shows three sagittal slices. 1, cerebellum anterior lobe; 4, posterior cingulate; 7, thalamus; 8, midbrain.

After ANCOVA analysis and *post hoc* test, we observed greater GM and WM volume losses in smokers with moderate nicotine dependence (moderate dependence smokers vs. non-smokers) compared to smokers with severe nicotine dependence (severe dependence smokers vs. non-smokers) (**Tables 2**, **3**). The primary atrophic GM areas (**Figure 1**) in the smokers with moderate nicotine dependence were the middle occipital gyrus (MOG), posterior cingulate (PC) cortex, cerebellum anterior lobe (CAL), precuneus, and caudate body (CB), although only some of these GM areas were found with significant atrophy in smokers with severe nicotine dependence (**Figure 1**). The atrophic WM areas were mainly located at PC, CAL and midbrain in the two nicotine dependence subgroups; however, the extent of brain tissue loss in most of these WM areas was also larger in the smokers with moderate nicotine dependence than in those with severe nicotine dependence (**Figure 2**).

**Table 2:** Regions of smaller Gray matter and White matter volumes in 23 mild/moderate nicotine dependence smokers than in 53 non-smokers.

Region	Side^a^	MNI^b^ coordinates [mm]			*Z*-score	Cluster size [voxels]
		*x*	*y*	*z*		
**Gray matter**						
Middle occipital gyrus (MOG)	L	−32	−69	12	2.29	265
Posterior cingulate (PC)	L	−6	−67.5	15	4.69	1196
Cerebellum anterior lobe (CAL)	L	−8	−52	−2	2.99	817
Precuneus	R	6	−65	19	2.12	134
	L	−17	−57	15	2.58	254
Caudate body (CB)	R	11	15	13	2.83	250
	L	−8	8	10	2.16	131
Insula	R	42	0	1.5	2.22	71
**White matter**						
Posterior cingulate (PC)	R	7.5	−60	10.5	2.65	249
Thalamus	R	6	−16.5	15	4.65	652
	L	−7	14	18	4.36	576
Cerebellum anterior lobe (CAL)	L	−4	−53	−2	2.13	82
Midbrain	R	4	−27	−1	1.72	46
	L	−7	−31	−1	1.68	43

*The threshold of significance was set at p < 0.05 (AlphaSim-corrected). ^a^L, left brain; R, right brain. ^b^MNI, Montreal Neurological Institute, All coordinates are provided in MNI space.*

**Table 3:** Regions of smaller Gray matter and White matter volumes in 30 severe nicotine dependence smokers than in 53 non-smokers.

Region	Side^a^	MNI^b^ coordinates [mm]			*Z*-score	Cluster size [voxels]
		*x*	*y*	*z*		
**Gray matter**						
Middle occipital gyrus (MOG)	L	−29	−70	−0	2.15	42
Posterior cingulate (PC)	R	25.5	−66	9	2.81	43
	L	−9	−61.5	12	2.04	67
Superior temporal gyrus (STG)	L	−46.5	−7.5	−9	2.82	68
Insula	L	−41	11	−8	2.09	107
**White matter**						
Posterior cingulate (PC)	R	10.5	−54	4.5	2.66	100
Thalamus	R	6	−22	4	2.92	89
	L	−12	−28	6	2.59	357
Cerebellum anterior lobe (CAL)	L	−7	−59	−2	2.36	41
Midbrain	L	−4.5	−39	−9	2.34	90

*The threshold of significance was set at p < 0.05 (AlphaSim-corrected). ^a^L, left brain; R, right brain. ^b^MNI, Montreal Neurological Institute, All coordinates are provided in MNI space.*

**FIGURE 3:**
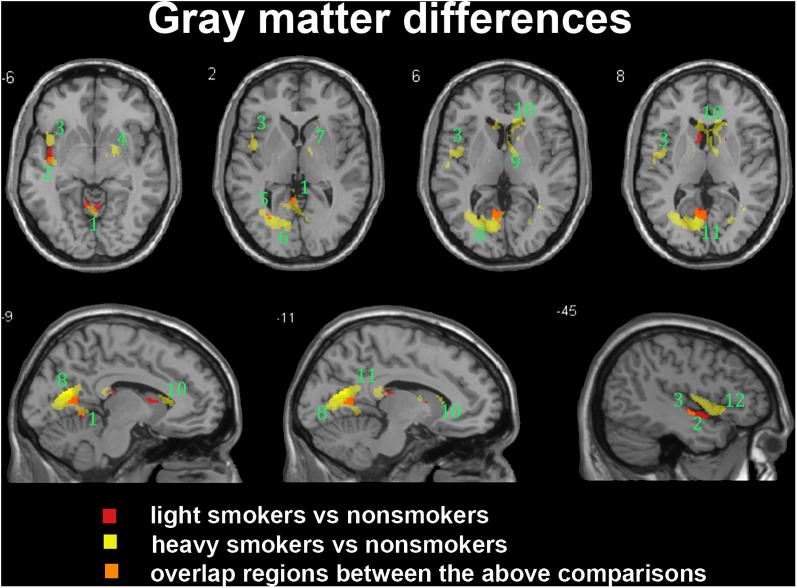
Regions with smaller gray matter volumes in 26 light smokers and 27 heavy smokers than in non-smokers. This image shows the overlap (orange) regions of the smaller gray matter in 26 light smokers (red) and 27 heavy smokers (yellow) than in 53 non-smokers. Overall thresholds of significance were set at *P* < 0.05 (AlphaSim corrected). The **(top row)** shows four axial slices, whereas the **(bottom row)** shows three sagittal slices. 1, cerebellum anterior lobe; 2, superior temporal gyrus; 3, insula; 4, putamen; 5, middle occipital gyrus; 6, lingual; 7, Pallidum; 8, posterior cingulate; 9, thalamus; 10, caudate body; 11, precuneus; 12, inferior frontal gyrus.

**FIGURE 4:**
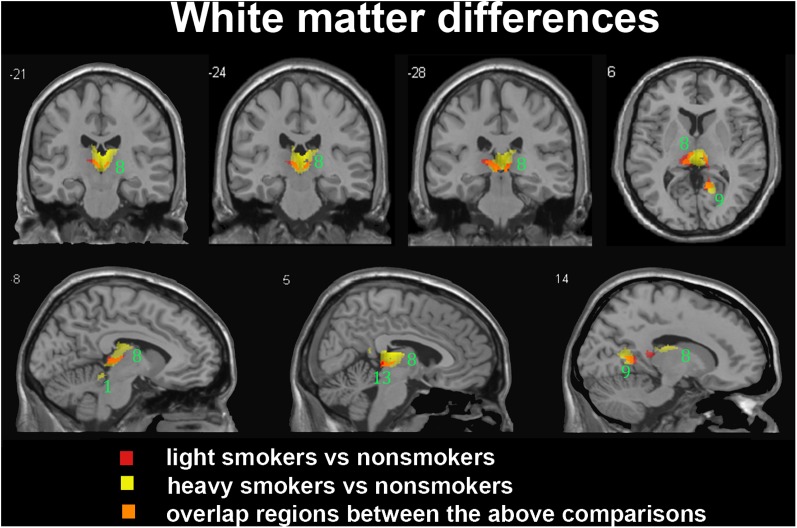
Regions with smaller white matter volumes in 26 light smokers and 27 heavy smokers than in non-smokers. This image shows the overlap (orange) regions of the smaller white matter in 26 light smokers (red) and 27 heavy smokers (yellow) than in 53 non-smokers. Overall thresholds of significance were set at *P* < 0.05 (AlphaSim corrected). The **(top row)** shows three coronal slices and one axial slice, whereas the **(bottom row)** shows three sagittal slices. 1, cerebellum anterior lobe; 8, thalamus; 9, posterior cingulate; 13, midbrain.

In the subgroup of light smokers, we found that the GM of superior temporal gyrus (STG), insula, MOG, PC, CAL, CB, and precuneus exhibited atrophy than non-smokers (**Figure 3**). However, these brain regions were severely atrophied in the subgroup of heavy smokers, and this subgroup also demonstrated other atrophic brain areas, such as the lingual gyrus, the middle temporal gyrus (MTG), the pallidum, the putamen, and the thalamus than non-smokers (**Figure 3**). The atrophic WM areas in the heavy smokers were also larger and more than observed in those within the light smokers group, especially in the PC, thalamus, and midbrain (**Figure 4**). No significant increases in the GM or WM volume of smokers were observed when compared with the non-smokers.

For between-group comparisons in smokers, we observed significant brain region atrophy in the subgroup of heavy smokers compared to light smokers, mainly in GM areas of precuneus, inferior frontal gyrus, insula, STG and cerebellum anterior lob, and in WM areas of cerebellum anterior lobe (**Table 4**). But an opposite trend was detected in the structural abnormalities caused by the severity of nicotine dependence, and smokers with moderate nicotine dependence were found with severe brain region atrophy in comparison to smokers with severe nicotine dependence, especially in GM areas of precuneus, superior and MTG, middle occipital gyrus, posterior cingulate and insula, and in WM areas of precuneus, posterior cingulate, cerebellum anterior lobe and midbrain (**Table 5**).

**Table 4:** Gray matter and White matter differences between light and heavy smoking amount groups.

Region	Side^a^	MNI coordinates^b^ [mm]			*Z*-score	Cluster size [voxels]
		*x*	*y*	*z*		
**Gray matter**						
Inferior frontal gyrus	R	55.5	18	4.5	4.32	1193
Insula	R	35	30	3	3.90	687
Superior temporal gyrus	R	59	0	3	3.40	485
Middle frontal gyrus	R	56	−33	4	3.18	154
Precuneus	L	−10.5	−51	34.5	4.41	751
Cerebellum anterior lobe	R	9	−52.5	−15	3.53	269
**White matter**						
Cerebellum anterior lobe	R	34.5	−36	34.5	3.84	460

*The threshold of significance was set at p < 0.05 (AlphaSim-corrected). ^a^L, left brain; R, right brain. ^b^MNI, Montreal Neurological Institute, All coordinates are provided in MNI space.*

**Table 5:** Gray matter and White matter differences between mild/moderate and severe nicotine dependence groups.

Region	Side^a^	MNI coordinates^b^ [mm]			*Z*-score	Cluster size [voxels]
		*x*	*y*	*z*		
**Gray matter**						
Insula	R	37.5	−4.5	13.5	3.79	597
Superior temporal gyrus	R	28.5	6	−30	3.18	1159
	L	−57	−24	−0	3.64	769
Middle temporal gyrus	R	44	−75	25	3.47	414
	L	−64.5	−9	−7.5	3.75	803
Precuneus	R	41	−75	39	3.14	365
	L	−7.5	−64.5	40.5	3.98	1275
Posterior cingulate	L	−3	−49.5	7.5	3.89	402
Middle occipital gyrus	R	30	−88.5	4.5	4.05	1336
	L	−21	−99	−1.5	3.11	59
Midbrain	R	15	−22.5	−15	3.42	82
	L	−16.5	−28.5	−3	3.52	42
Cerebellum anterior lobe	R	7.5	−61.5	−16.5	3.65	342
**White matter**						
Precuneus	R	22.5	−81	43.5	3.68	521
	L	−5	−81	48	2.65	50
Posterior cingulate	L	−4.5	−46.5	4.5	3.75	137
Cerebellum anterior lobe	R	36	−34.5	−34.5	3.75	268
Midbrain	R	15	−21	−15	3.53	102

*The threshold of significance was set at p < 0.05 (AlphaSim-corrected). ^a^L, left brain; R, right brain. ^b^MNI, Montreal Neurological Institute, All coordinates are provided in MNI space.*

For correlational analysis, most between-group (i.e., light vs. heavy smokers; moderate vs. severe nicotine dependence smokers) abnormal regions demonstrated significant correlations with pack-years or FTND score (**Table 6**).

**Table 6:** Correlational analysis between abnormal brain regions and clinical scores in smokers.

Clinical score	Brain region	*r* value	*P*-value
Pack-years	R.IFG	−0.401	0.031
	R.insula	−0.367	0.009
	R.MFG	−0.324	0.043
	L.PRE	−0.298	0.028
	R.CAL (WM)	−0.358	0.036
FTND score	R.insula	0.368	0.045
	R.STG	0.291	0.033
	L.STG	0.297	0.039
	L.MTG	0.431	0.008
	L.PRE	0.398	0.013
	L.PCC	0.401	0.018
	R.MOG	0.384	0.024
	R.PRE	0.336	0.034
	R.CAL	0.359	0.039

*R., right; L., left; IFG, inferior frontal gyrus; MFG, middle frontal gyrus; PRE, precuneus; CAL, Cerebellum anterior lobe; (WM), white matter; STG, superior temporal gyrus; MTG, middle temporal gyrus; PCC, posterior cingulate cortex; MOG, middle occipital gyrus.*

## Discussion

Many studies have found that smokers exhibit structural changes in some brain areas in comparison to non-smokers (Karama et al., 2015; Peng et al., 2015; Prom-Wormley et al., 2015). Our previous study went further to discover that brain tissue atrophy becomes more obvious with the associated smoking amount (pack-years) (Peng et al., 2015), which demonstrates that pack-years is an effective indicator of smoking-related structural damages in the brain. In this study, we found that severe nicotine dependence smokers demonstrated less brain atrophy than moderate nicotine dependence smokers, interestingly illustrating a distinct change trend in comparison to the findings from pack-years.

Tobacco consumption and smoking dependence represent different aspects regarding smokers, and there weren’t any potential inherent relationships between them. A previous study found that nicotine craving may not necessarily attenuate with the number of years one has smoked tobacco (John et al., 2003). In the current study, we found 12 light tobacco consumption smokers with severe nicotine dependence and 9 heavy tobacco consumption smokers with moderate nicotine dependence. The average age of the 12 light tobacco consumption smokers was 27.08 ± 3.48 years, while that of the 9 heavy smokers with moderate nicotine dependence was 34.78 ± 3.83 years, and their ages were significantly different (*p* < 0.001). However, there were no significant differences between the ages at the time of first smoking in these smokers (*p* = 0.722). This may imply that smokers with severe nicotine dependence may not smoke too long and only had few years’ smoking history. In addition, the potential genetic changes in the nicotinic receptor expression with advancing age may also account for the moderate nicotine dependence found in heavy smokers. Notably, the Chinese tobacco control policy remains lenient, and the number of cigarettes consumed per day in the severe dependence group is significantly higher than in the moderate dependence group in our study. However, in countries such as the United Kingdom and the United States where anti-smoking policies stand firm, the number of cigarettes per day may not be a reliable indicator of nicotine dependence as there might not be any substantial differences in nicotine dependence outcomes. Based on the previous study, it was reported that smokers’ true level of nicotine dependence could be underestimated by only relying on the number of cigarettes per day (Fagerstrom, 2003), which may lead to bias in the nicotine dependence related outcomes.

For the correlation analysis, the volume of several regions including the right inferior frontal gyrus, the right insula, the right middle frontal gyrus, the left precuneus and the right cerebellum anterior lobe were significantly correlated with the pack-years. Moreover, the volume of the right insula, the right/left STG, the left MTG, the left/right precuneus, the left posterior cingulate cortex, the right middle occipital gyrus and the right cerebellum anterior lobe were positively correlated with the FTND score. Interestingly, the right insula and the left precuneus were particularly found significant correlations with both pack-years and FTND score, implying the nicotine dependence and smoking consumption may have some internal interactions on the brain volume. The precuneus is known as an important part of the default mode network (Fransson and Marrelec, 2008; Utevsky et al., 2014), which is related to the self-consciousness, episodic memory retrieval, visuo-spatial imagery and self-related mental representations during rest. The precuneus has been reported to be the cortical core for neuroplastic changes related to drug dependence (Courtney et al., 2014), and its cortical thinning has been found to have a significant correlation with pack-years (Karama et al., 2015). Our results consistently consolidate previous findings on precuneus in smokers and further demonstrate that both nicotine dependence and smoking consumption display influences on the left precuneus. The insula is referred to as the core part of salience network (Seeley et al., 2007), which is associated with the switch between internal and external stimuli, consequently dictating behavioral outcomes (Sridharan et al., 2008; Menon, 2011). In a previous study, the right insula was found with abnormal functional and structural connectivities in young adult smokers, and connections between right insula and anterior cingulate cortex were correlated with both FTND and pack-years (Li et al., 2016). Our results replicate the reported interactive effects of nicotine dependence and smoking consumption regarding the right insula, implying that the volume of right insula also reflects the smoking neural alterations. In addition, the right inferior frontal gyrus and middle frontal gyrus have been reported to play roles in attentive control and reorientation (Hampshire et al., 2010; Japee et al., 2015), which may be directly associated with smoking behavior and their volumes were found negative correlations with the pack-years. Moreover, the MTG and posterior cingulate cortex (Wang et al., 2017), the STG (Ettinger et al., 2009) and the middle occipital gyrus (Liu et al., 2014) have been reported to be associated with the nicotine dependence, and their volumes were also found significantly correlated with the FTND score in this study. Specifically, the cerebellum anterior lobe, which is part of the cerebellum, is traditionally thought to be responsible for motor control and has particularly been focused on in smokers (Kühn et al., 2012; Huang et al., 2017). The cerebellum anterior lobe has been found to display abnormality in smokers by resting state fMRI (Chu et al., 2014), and our results further complements to the understanding of the role of cerebellum anterior lobe in smokers.

In this study, the effects of nicotine dependence and tobacco consumption on brain structural changes are compared, and nicotine dependence was found with distinct atrophy patterns on the brain volume in contrast to the findings of tobacco consumption. It is very interesting to find that mild nicotine dependence smokers displayed more structural alternations than the heavy nicotine dependence smokers. We speculate this phenomenon may be related to the intensified neuroplasticity, in other words, when the brain experiences more cigarettes within a certain period of time, the brain becomes accustomed to the adverse effects of smoking and may adjust itself against brain atrophy. Another possible reason is that severe nicotine dependence may accompany with intense pleasure, which may act as a protective role for the brain volume. Furthermore, a previous study (Shen et al., 2016) also reported the similar phenomenon, and smokers with mild dependence were found with weaker connectivity strength than those with severe dependence, and the connectivity strengths in severe dependence smokers were even in the range of healthy controls. Notably, the nicotine dependence may be influenced by some other factors, such as nicotinic receptor expression, socioeconomic status, occupation and family smoking history, which are hard to be controlled in most studies. Taken together, further study should be designed to precisely delineate of the interactions between nicotine dependence and brain structure.

In clinical interventions, physicians often use the FTND as a reference marker to determine the dosage of smoking cessation medication, such as nicotine replacement therapy (NRT). The more severe the dependence, the more difficult to quit, and the logically more easier to relapse, in other words, the more serious the tobacco dependence is, the more likely that smokers will benefit from smoking cessation interventions – especially from the perspectives of pharmacotherapeutic aspects. The first authoritative and systematic report on the health hazards of smoking in China edited by the Ministry of Health of the People’s Republic of China, reported that smokers who exhibit no or little tobacco dependence could potentially quit smoking with sole implementation of willpower, as well as with the professional assistances from doctors, while severe dependence smokers often require stronger pharmacological agents’ interventions to assist in smoking cessation (Ministry of Health of the People’s Republic of China, 2012). However, based on our results, we speculate that the choice of treatment should also combine with the extent of damage caused by cumulative smoking amount (as related to pack-years). Some smokers with moderate nicotine dependence may already have severe brain damages given that they had smoked large amounts for a long time. In China – one of the world’s largest tobacco-producing and consuming countries – most smokers only exhibited mild nicotine dependence (Yang et al., 2011). The average FTND score was 2.89 (95% confidence interval [CI]: 2.77–3.01) among all current smokers, and even daily smokers did not exceed an FTND score of 4.00 (3.49; 95% CI: 3.35–3.63). However, most smokers presented with more than 10 years of smoking history, and smokers with a smoking history of more than 20 years, or even longer, were not rare either (Gruder et al., 2013). Even if they only smoked a few cigarettes a day, the total smoking amount (pack-years) is large, implying that their health states have been significantly impaired. Specially, smoking is still prevalent and tolerated in many public places in China, and considering China’s fairly lax anti-smoking policies, it becomes hard to avoid or mitigate smoking without the assistance of effective medical interventions, even in smokers without severe nicotine dependence. Therefore, it is highly encouraged that physicians should give more active smoking cessation interventions to these smokers with mild nicotine dependence but large tobacco consumption.

Several limitations should be also mentioned. First, the CO levels in expired air for smokers and nonsmokers were not evaluated before the MRI scan, which is an objective index for smoking behavior. Second, regarding the white matter, the volume just stands for a type of macroscopic features, and the microscopic features of white matter have recently become the research focus with diffusion tensor imaging, which we will explore in future research.

## Conclusion

This current study confirms that brain volume atrophy appears to be more serious in smokers with higher levels of cigarette consumption and in smokers with moderate nicotine dependence, and nicotine dependence displayed contrary patterns on the brain volume of smokers compared with tobacco consumption. Our results indicated that the combinations of the FTND, pack-years and MRI findings could help to comprehensively evaluate the cumulative influences of smoking on smokers and to well understand the roles of affected brain regions, which finally promotes the development of appropriate medical interventions for smokers.

## Ethics Statement

This study was carried out in accordance with the recommendations of the ethics committee of Beijing Chaoyang Hospital with written informed consent from all subjects. All subjects gave written informed consent in accordance with the Declaration of Helsinki. The protocol was approved by the ethics committee of Beijing Chaoyang Hospital.

## Author Contributions

PP, BJ, and TJ made substantial contributions to the conception, design, analysis and interpretation of data, and drafted the manuscript. BJ and HL made contributions to the revision of the manuscript. ML, Y-RT, S-LC, and NVH-L made contributions to the data acquisition. All authors read and approved the final manuscript.

## Conflict of Interest Statement

The authors declare that the research was conducted in the absence of any commercial or financial relationships that could be construed as a potential conflict of interest.

## Footnotes

1 http://www.fil.ion.ucl.ac.uk/spm

2 http://www.fil.ion.ucl.ac.uk/spm/doc/spm8_manual.pdf

3 http://rfmri.org/dpabi

## References

[B1] Ashburner J. Friston K. J. (2000). Voxel-based morphometry – the methods. 11(6 Pt 1), 805–821. 10.1006/nimg.2000.0582 10860804

[B2] Brody A. L. (2006). Functional brain imaging of tobacco use and dependence. 40 404–418. 10.1016/j.jpsychires.2005.04.012 15979645 PMC2876087

[B3] Cena H. Tesone A. Niniano R. Cerveri I. Roggi C. Turconi G. (2013). Prevalence rate of Metabolic Syndrome in a group of light and heavy smokers. 5:28. 10.1186/1758-5996-5-28 23721527 PMC3673853

[B4] Chu S. Xiao D. Wang S. Peng P. Xie T. He Y. (2014). Spontaneous brain activity in chronic smokers revealed by fractional amplitude of low frequency fluctuation analysis: a resting state functional magnetic resonance imaging study. 127 1504–1509. 24762597

[B5] Courtney K. E. Ghahremani D. G. London E. D. Ray L. A. (2014). The association between cue-reactivity in the precuneus and level of dependence on nicotine and alcohol. 141 21–26. 10.1016/j.drugalcdep.2014.04.026 24880692 PMC4166553

[B6] Ding X. Lee S. W. (2013). Changes of functional and effective connectivity in smoking replenishment on deprived heavy smokers: a resting-state FMRI study. 8:e59331. 10.1371/journal.pone.0059331 23527165 PMC3602016

[B7] Durazzo T. C. Insel P. S. Weiner M. W. Alzheimer Disease Neuroimaging Initiative (2012). Greater regional brain atrophy rate in healthy elderly subjects with a history of cigarette smoking. 8 513–519. 10.1016/j.jalz.2011.10.006 23102121 PMC3484322

[B8] Durazzo T. C. Meyerhoff D. J. Nixon S. J. (2013). Interactive effects of chronic cigarette smoking and age on hippocampal volumes. 133 704–711. 10.1016/j.drugalcdep.2013.08.020 24051060 PMC3870586

[B9] Duriez Q. Crivello F. Mazoyer B. (2014). Sex-related and tissue-specific effects of tobacco smoking on brain atrophy: assessment in a large longitudinal cohort of healthy elderly. 6:299. 10.3389/fnagi.2014.00299 25404916 PMC4217345

[B10] Eklund A. Nichols T. E. Knutsson H. (2016). Cluster failure: Why fMRI inferences for spatial extent have inflated false-positive rates. 113 7900–7905. 10.1073/pnas.1602413113 27357684 PMC4948312

[B11] Ettinger U. Williams S. C. Patel D. Michel T. M. Nwaigwe A. Caceres A. (2009). Effects of acute nicotine on brain function in healthy smokers and non-smokers: estimation of inter-individual response heterogeneity. 45 549–561. 10.1016/j.neuroimage.2008.12.029 19159693

[B12] Fagerstrom K. (2003). Time to first cigarette; the best single indicator of tobacco dependence? 59 91–94. 14533289

[B13] Feng D. Yuan K. Li Y. Cai C. Yin J. Bi Y. (2016). Intra-regional and inter-regional abnormalities and cognitive control deficits in young adult smokers. 10 506–516. 10.1007/s11682-015-9427-z 26164168

[B14] Fransson P. Marrelec G. (2008). The precuneus/posterior cingulate cortex plays a pivotal role in the default mode network: evidence from a partial correlation network analysis. 42 1178–1184. 10.1016/j.neuroimage.2008.05.059 18598773

[B15] Fritz H. C. Wittfeld K. Schmidt C. O. Domin M. Grabe H. J. Hegenscheid K. (2014). Current smoking and reduced gray matter volume-a voxel-based morphometry study. 39 2594–2600. 10.1038/npp.2014.112 24832823 PMC4207339

[B16] Gruder C. L. Trinidad D. R. Palmer P. H. Xie B. Li L. Johnson C. A. (2013). Tobacco smoking, quitting, and relapsing among adult males in Mainland China: the China Seven Cities Study. 15 223–230. 10.1093/ntr/nts116 22581939 PMC3611989

[B17] Hampshire A. Chamberlain S. R. Monti M. M. Duncan J. Owen A. M. (2010). The role of the right inferior frontal gyrus: inhibition and attentional control. 50 1313–1319. 10.1016/j.neuroimage.2009.12.109 20056157 PMC2845804

[B18] Hoogendam Y. Y. van der Geest J. N. van der Lijn F. van der Lugt A. Niessen W. J. Krestin G. P. (2012). Determinants of cerebellar and cerebral volume in the general elderly population. 33 2774–2781. 10.1016/j.neurobiolaging.2012.02.012 22405042

[B19] Huang P. Shen Z. Wang C. Qian W. Zhang H. Yang Y. (2017). Altered white matter integrity in smokers is associated with smoking cessation outcomes. 11:438. 10.3389/fnhum.2017.00438 28912702 PMC5582085

[B20] Ikram M. A. Vrooman H. A. Vernooij M. W. van der Lijn F. Hofman A. van der Lugt A. (2008). Brain tissue volumes in the general elderly population. The Rotterdam Scan Study. 29 882–890. 10.1016/j.neurobiolaging.2006.12.012 17239994

[B21] Janjigian Y. Y. McDonnell K. Kris M. G. Shen R. Sima C. S. Bach P. B. (2010). Pack-years of cigarette smoking as a prognostic factor in patients with stage IIIB/IV nonsmall cell lung cancer. 116 670–675. 10.1002/cncr.24813 20029977 PMC2815173

[B22] Japee S. Holiday K. Satyshur M. D. Mukai I. Ungerleider L. G. (2015). A role of right middle frontal gyrus in reorienting of attention: a case study. 9:23. 10.3389/fnsys.2015.00023 25784862 PMC4347607

[B23] Jasinska A. J. Zorick T. Brody A. L. Stein E. A. (2014). Dual role of nicotine in addiction and cognition: a review of neuroimaging studies in humans. 84 111–122. 10.1016/j.neuropharm.2013.02.015 23474015 PMC3710300

[B24] John U. Meyer C. Hapke U. Rumpf H. J. Schumann A. Adam C. (2003). The Fagerstrom test for nicotine dependence in two adult population samples-potential influence of lifetime amount of tobacco smoked on the degree of dependence. 71 1–6. 10.1016/S0376-8716(03)00038-3 12821200

[B25] Karama S. Ducharme S. Corley J. Chouinard-Decorte F. Starr J. M. Wardlaw J. M. (2015). Cigarette smoking and thinning of the brain’s cortex. 20 778–785. 10.1038/mp.2014.187 25666755 PMC4430302

[B26] Kühn S. Romanowski A. Schilling C. Mobascher A. Warbrick T. Winterer G. (2012). Brain grey matter deficits in smokers: focus on the cerebellum. 217 517–522. 10.1007/s00429-011-0346-5 21909705

[B27] Li Y. Yuan K. Cai C. Feng D. Yin J. Bi Y. (2015). Reduced frontal cortical thickness and increased caudate volume within fronto-striatal circuits in young adult smokers. 151 211–219. 10.1016/j.drugalcdep.2015.03.023 25865908

[B28] Li Y. Yuan K. Guan Y. Cheng J. Bi Y. Shi S. (2016). The implication of salience network abnormalities in young male adult smokers. 11 943–953. 10.1007/s11682-016-9568-8 27437925

[B29] Liu J. Claus E. D. Calhoun V. D. Hutchison K. E. (2014). Brain regions affected by impaired control modulate responses to alcohol and smoking cues. 75 808–816. 10.15288/jsad.2014.75.808 25208199 PMC4161701

[B30] McEvoy J. W. Blaha M. J. DeFilippis A. P. Lima J. A. Bluemke D. A. Hundley W. G. (2015). Cigarette smoking and cardiovascular events: role of inflammation and subclinical atherosclerosis from the MultiEthnic Study of Atherosclerosis. 35 700–709. 10.1161/ATVBAHA.114.304562 25573855 PMC4404404

[B31] Menon V. (2011). Large-scale brain networks and psychopathology: a unifying triple network model. 15 483–506. 10.1016/j.tics.2011.08.003 21908230

[B32] Ministry of Health of the People’s Republic of China (2012). Beijing: People’s medical publishing house, 246. 10.1111/crj.12393

[B33] Peng P. Wang Z. Jiang T. Chu S. Wang S. Xiao D. (2015). Brain-volume changes in young and middle-aged smokers: a DARTEL-based voxel-based morphometry study. 11 621–631. 10.1111/crj.12393 26404024

[B34] Pennington D. L. Durazzo T. C. Schmidt T. P. Abé C. Mon A. Meyerhoff D. J. (2015). Alcohol use disorder with and without stimulant use: brain morphometry and its associations with cigarette smoking, cognition, and inhibitory control. 10:e0122505. 10.1371/journal.pone.0122505 25803861 PMC4372577

[B35] Power M. C. Deal J. A. Sharrett A. R. Jack C. R. Jr. Knopman D. Mosley T. H. (2015). Smoking and white matter hyperintensity progression: the ARIC-MRI Study. 84 841–848. 10.1212/WNL.0000000000001283 25632094 PMC4345648

[B36] Prom-Wormley E. Maes H. H. Schmitt J. E. Panizzon M. S. Xian H. Eyler L. T. (2015). Genetic and environmental contributions to the relationships between brain structure and average lifetime cigarette use. 45 157–170. 10.1007/s10519-014-9704-4 25690561 PMC4348348

[B37] Seeley W. W. Menon V. Schatzberg A. F. Keller J. Glover G. H. Kenna H. (2007). Dissociable intrinsic connectivity networks for salience processing and executive control. 27 2349–2356. 10.1007/s00213-016-4262-5 17329432 PMC2680293

[B38] Shen Z. Huang P. Qian W. Wang C. Yu H. Yang Y. (2016). Severity of dependence modulates smokers’ functional connectivity in the reward circuit: a preliminary study. 233 2129–2137. 10.1007/s00213-016-4262-5 26955839

[B39] Sridharan D. Levitin D. J. Menon V. (2008). A critical role for the right fronto-insular cortex in switching between central-executive and default-mode networks. 105 12569–12574. 10.1073/pnas.0800005105 18723676 PMC2527952

[B40] Utevsky A. V. Smith D. V. Huettel S. A. (2014). Precuneus is a functional core of the default-mode network. 34 932–940. 10.1523/JNEUROSCI.4227-13.2014 PMC3891968 24431451

[B41] Wang C. Shen Z. Huang P. Qian W. Yu X. Sun J. (2017). Altered spontaneous activity of posterior cingulate cortex and superior temporal gyrus are associated with a smoking cessation treatment outcome using varenicline revealed by regional homogeneity. 11 611–618. 10.1007/s11682-016-9538-1 26960945

[B42] Wang C. Xu X. Qian W. Shen Z. Zhang M. (2015). Altered human brain anatomy in chronic smokers: a review of magnetic resonance imaging studies. 36 497–504. 10.1007/s10072-015-2065-9 25577510

[B43] World Health Organization [WHO] (1992). Geneva: World Health Organization. 10.1093/ntr/ntr040

[B44] Yang T. Shiffman S. Rockett I. R. Cui X. Cao R. (2011). Nicotine dependence among Chinese city dwellers: a population-based cross-sectional study. 13 556–564. 10.1093/ntr/ntr040 21454911

[B45] Yu R. Zhao L. Tian J. Qin W. Wang W. Yuan K. (2013). Regional homogeneity changes in heavy male smokers: a resting-state functional magnetic resonance imaging study. 18 729–731. 10.1111/j.1369-1600.2011.00359.x 21812873

[B46] Yuan C. Morales-Oyarvide V. Babic A. Clish C. B. Kraft P. Bao Y. (2017). Cigarette smoking and pancreatic cancer survival. 35 1822–1828. 10.1200/JCO.2016.71.2026 28358654 PMC5455596

